# Mate Choice in Adult Female Bengalese Finches: Females Express Consistent Preferences for Individual Males and Prefer Female-Directed Song Performances

**DOI:** 10.1371/journal.pone.0089438

**Published:** 2014-02-18

**Authors:** Jeffery L. Dunning, Santosh Pant, Aaron Bass, Zachary Coburn, Jonathan F. Prather

**Affiliations:** Program in Neuroscience, Department of Zoology and Physiology, University of Wyoming, Laramie, Wyoming, United States of America; Claremont Colleges, United States of America

## Abstract

In the process of mate selection by female songbirds, male suitors advertise their quality through reproductive displays in which song plays an important role. Females evaluate the quality of each signal and the associated male, and the results of that evaluation guide expression of selective courtship displays. Some studies reveal broad agreement among females in their preferences for specific signal characteristics, indicating that those features are especially salient in female mate choice. Other studies reveal that females differ in their preference for specific characteristics, indicating that in those cases female evaluation of signal quality is influenced by factors other than simply the physical properties of the signal. Thus, both the physical properties of male signals and specific traits of female signal evaluation can impact female mate choice. Here, we characterized the mate preferences of female Bengalese finches. We found that calls and copulation solicitation displays are equally reliable indicators of female preference. In response to songs from an array of males, each female expressed an individual-specific song preference, and those preferences were consistent across tests spanning many months. Across a population of females, songs of some males were more commonly preferred than others, and females preferred female-directed songs more than undirected songs, suggesting that some song features are broadly attractive. Preferences were indistinguishable for females that did or did not have social experience with the singers, indicating that female preference is strongly directed by song features rather than experiences associated with the singer. Analysis of song properties revealed several candidate parameters that may influence female evaluation. In an initial investigation of those parameters, females could be very selective for one song feature yet not selective for another. Therefore, multiple song parameters are evaluated independently. Together these findings reveal the nature of signal evaluation and mate choice in this species.

## Introduction

In the context of mate selection by female songbirds, male suitors seek to attract female mates through their song performances and other signals such as plumage or reproductive displays. Females detect those signals and use that sensory information to form a subjective evaluation of the quality of each signal and the associated male. The results of that cognitive behavior then guide the female’s expression of selective motor responses such as approaching only the preferred male, responding with courtship displays, performing some operant task, or assuming specific postures to initiate copulation [1], [2], [3], [4], [5], [6]. Some studies reveal that females express broad agreement in their preferences for specific signal characteristics, indicating that those features are especially salient in female mate choice [2], [3], [7], [8], [9], [10]. Interestingly, other studies reveal that individual females differ in their preference for specific characteristics, indicating that the female’s evaluation can be influenced by individual-specific features of perception rather than simply the physical properties of the signal [11], [12]. Thus, both the physical properties of reproductive signals and the individual-specific nature of the cognitive behaviors through which those signals are evaluated can have a profound impact on female mate choice.

Female songbirds are an excellent model in which to investigate how the features of reproductive signals and the individual-specific features of signal evaluation combine to influence a female bird’s choice of a specific male suitor. In many songbird species, including the Bengalese finches (BFs) studied here, song is used by males to attract female mates, and males sing but females do not [13], [14]. Yet despite their inability to sing, female songbirds are equal or superior to males in their ability to perceive the fine details that distinguish the songs of different species or the songs of different males within a species [15], [16], [17]. In tests of the degree to which female mate preference is influenced by song as opposed to visual characteristics such as beak color, song plays the more important role in the species where that has been tested [18], [19]. The importance of song in shaping female mate preference is especially clear in cases when females perform a copulation solicitation display (CSD) in response to song, briefly adopting a posture reminiscent of mammalian lordosis that facilitates male reproductive access. In fact, song is such a powerful influence on female mate choice that females will perform CSDs in response to songs played through a speaker, even with no male physically present [2], [5]. Therefore, CSDs are a well-established and easily interpreted means of assessing female songbird mate choice, and quantifying their expression can be used to identify songs that an individual female finds especially attractive. Features of those songs can be compared against the features of songs that she finds less attractive to identify parameters that may be especially salient in shaping the preferences of individual females. The BFs studied here provide an important advantage in that regard, as the songs of an individual male vary in their note sequence within and across songs [13]. That natural variation makes BF songs more complex than those of other commonly studied species, providing enhanced resolution in efforts to define which song parameters are most salient in shaping female preference. Furthermore, BFs fare especially well and display their full spectrum of courtship and reproductive behaviors in the laboratory. These features make BFs excellent candidates for comprehensive investigation of the mechanisms through which females evaluate the quality of male signals and use that information to guide mate selection.

Previous experiments have provided some insight into the nature of female mate choice and the relation between that preference and the features of male song. Specifically, studies of other species have revealed that females can recognize individual males by their songs, and female preferences for the songs of specific males are consistent across time and trials [20]. Comparative approaches have also identified features of male song that are important in directing the preference of females of different species. Female mate choice has been linked to many challenging aspects of song performance including long duration [7], [10], loud amplitude [21], combinations of large bandwidth and rapid trilling [3], the inclusion of specific syllables [8], [9], [22], and the complexity of song content or sequencing [5], [23], [24], [25], [26], [27]. Thus, studies of other species reveal that female preferences for specific songs and therefore specific males are associated with specific song parameters.

In the few studies that have investigated mate selection in BFs, the relation between male song and female preference is much less defined. Some studies have identified the number of different song notes [5] and the complexity of note sequence as contributors to female preference [28]. However other work from the same group reveals little or no relation between note sequence complexity and female preference [25], [29]. This lack of clarity leaves open fundamental questions regarding the nature of female BF preference and its relation to the parameters of male song. Specifically, it remains unresolved whether variability in female BF mate preference emerges because song complexity plays little or no role in directing female preference, or whether complexity is influential in some cases but females are highly variable in the nature of the song features that each female prefers. Relevant to that consideration, males change many song parameters when they sing either alone (“undirected”) or in the presence of a female (“directed”) [30], [31], and those changes are functionally relevant, as females typically prefer songs performed in the directed condition [32]. The distributed nature of changes in the directed condition suggests that one parameter is likely not the sole determinant of female BF preference. Therefore, essential next steps toward understanding the basis of signal evaluation and mate selection in this species are to determine whether song preferences are consistent within individual females, whether song preferences are consistent across individuals, and whether multiple song parameters can be evaluated independently of one another.

In this study, we characterized the song preferences of adult female Bengalese finches. In Phase 1 of this study, we address the hypothesis that female BF mate choice is evident not only in the expression of copulation solicitation displays but also in the number of calls that the female performs during exposure to song stimuli. We predict that if CSDs and calls are equally reliable indicators of BF mate choice [33], then we should observe a strong correlation between each female’s most-preferred song stimulus as revealed by each indicator. In Phase 2, we address the hypothesis that female BF mate preference is directed by the properties of the song stimulus itself and does not require experience with the associated singer. We predict that although the identity of the most preferred male may vary across different females, each female BF’s preference for her most-preferred male will be consistent across time and repeated tests. We further predict that if preference is dictated by the properties of the song signal itself, then preferences will be broadly similar between females that have interacted with the associated male singers and other females that have not interacted with those males. If supported by the data, a finding that female mate preferences are consistent within individuals and do not require specific aspects of past experience with the associated singer (e.g., mate, cage partner, etc.) would vastly facilitate subsequent investigations of the neural circuitry through which songs are perceived and evaluated in this species. Finally, we also address the hypothesis that female BFs prefer directed songs more than undirected song. We predict that female BFs will prefer directed song, and that subtle differences exist between undirected and directed performances by one and the same male (for a similar investigation in zebra finches, see also [30], [32]). Such a result would reveal that those subtle differences are functionally significant, and those data could guide subsequent identification of one or more song features that are especially salient in affecting female BF song preference. Thus, the results of this study will reveal the nature of female BF signal evaluation and mate choice. Because we have collected these data in a species in which it is relatively easy to raise individuals under carefully controlled conditions and to collect neurophysiological data during behavioral tests, these insights will set the stage for additional investigation of the developmental processes through which individual-specific preferences emerge and the neural circuits through which sensory signals are evaluated and used to direct mate choice.

## Methods

### Ethics Statement

We performed all experiments using adult (age > 120 days post-hatch) male and female Bengalese finches (BF, *Lonchura striata domestica*) obtained from our breeding colony or from a commercial breeder. All procedures in this study were approved by the University of Wyoming Animal Care and Use Committee, and procedures were in compliance with recommendations from that group and state and federal regulations governing the housing and use of songbirds.

### Care and Handling of Experimental Subjects

Prior to experimentation, we identified males by their song performance, and we identified females by the presence of calls but the absence of song over three or more days of continuous recording. We housed animals in a colony setting prior to the experimental protocols detailed below, and throughout the experiment we maintained the 15:9 light:dark photoperiod used in our colony. Before beginning behavioral tests, we removed female subjects from the colony and placed them in sound attenuating chambers in all-female groups of no more than 5 birds for a minimum of 3 days to isolate those birds and prevent them from hearing song or interacting with male birds [26]. During the period when the birds were tested, subjects remained in these isolated groups, but we kept no bird in this condition for more than 14 consecutive days before returning them to the colony for a minimum of 3 days. At the time of each behavioral test, we moved the female subject from this isolation chamber (41 cm×31 cm×24 cm) to the chamber where we performed our behavioral tests (41 cm×33 cm×25 cm). We allowed at least 30 minutes to pass between moving the bird into the behavioral testing chamber and the beginning of testing [34]. In addition, we began testing only after the bird’s behavior indicated that it had become comfortable in its new surroundings (e.g., feeding, grooming; tests typically began 30 min after transition).

### Creation and Presentation of Song Stimuli

We recorded the songs of 8 individual BF males for at least 24 hours (range 24-36 hours) in a sound attenuation chamber in which we provided seed and water *ad libitum*. We monitored vocal behavior using a microphone (Shure model SM57) positioned immediately adjacent to the bird’s cage (41 cm×31 cm×24 cm) and custom software to continually record sounds and save them onto a computer hard drive using custom software (Sound Analysis Pro; songs were bandpass filtered 300 – 10000 Hz; Matlab software). We recorded each male twice in the sound attenuating chamber, once while alone (“undirected”) and once in the presence of a female (“directed”). In the behavioral tests described in the Results, we did not use any of the females that were used to evoke directed song, and we were careful to ensure that none of the songs in the directed condition contained female calls or other cage noise.

We used recordings of undirected song to represent each of the 8 males in tests of female preference for one among many males. We randomly selected 5 songs performed by each male and screened them only by duration so that the total duration of all 5 songs together was approximately 60 sec (duration of individual songs: 10.18±0.36 sec). We concatenated the songs of each male into one aggregate song stimulus containing all 5 songs with 700 ms of silence inserted between each song. None of these individual or concatenated songs had introductory notes, as introductory notes were either absent in the original song performance or we excluded them when individual songs were assembled into the aggregate song stimulus. Thus, we represented each male by 1 aggregate song stimulus containing a string of songs collected in the undirected condition and comprising approximately 60 sec of sound and silence together (duration of aggregate undirected song stimuli: 56.88±1.77 sec).

We also repeated this process of song selection and assembly using a set of directed songs and another set of undirected songs performed by the same set of 8 males, and we used the resulting set of stimuli to test female responses to directed versus undirected song performances (duration of aggregate undirected song stimuli: 60.63±3.77 sec; duration of aggregate directed song stimuli: 61.63±3.78 sec; ratio of directed:undirected duration for each male 1.02±0.01; durations were indistinguishable in each condition: paired t-test, t(7)  = 1.59, p = 0.15). We normalized the amplitude of all song stimuli such that each song had the same standard deviation of amplitude. During experimentation, we housed females individually in a cage inside a sound attenuation chamber (additional details below) and we presented song stimuli at 70 dB through a speaker located 5 to 22 cm away (70 dB measured 13 cm from the speaker, distance from speaker varied according to the bird’s location in the cage). We played song stimuli in a random sequence with an interval of 20 to 25 sec (randomly specified by computer) of silence between each song, and we presented each aggregate song once per test. In an attempt to prevent overexposure and habituation to the stimuli, we did not test any bird more than two times in a given day.

### Quantifying Female Responses to Song Stimuli

We measured each female’s behavioral responses to presentation of song stimuli by playing song through a speaker (Sound Acoustics) and viewing images and listening to audio captured through a camera (General Electric model 45231) located outside the bird’s cage but inside the sound attenuating chamber. Using these tools, we could clearly see and hear the female subject throughout experimentation. We scored all responses in real time, and we kept a recording of each test on video tape in the event that it was necessary to review the data to ensure accuracy.

During song presentation, females responded with many behaviors including the calls and copulation solicitation displays quantified in the Results. A copulation solicitation display is the most easily interpreted indicator of female mate choice. We recognized the occurrence of a CSD when the bird adopted a posture with its head down, its tail feathers slightly raised and often slightly fanned, and its wings either spread out to the side or slightly above the height of the bird’s back [35]. Females typically held that posture for approximately 2 seconds. We detected no clear gradation of CSD expression in our birds’ behavior, therefore we did not attempt to score subtle features of CSD gradation as others have done [35], [36]. Because our microphone was close to the bird’s cage, we could detect calls of even relatively low amplitude (e.g., when the bird was not facing the microphone), and we could often visually confirm the occurrence of a call as a brief opening of the bird’s beak. Other behaviors included vigorous perch hopping and occasional beak swiping or wing flaps [26]. Most females exhibited perch hopping at very high frequency across many or all treatment conditions [26]. Females expressed wing flaps less commonly, but they occurred across many or all treatment conditions, and it was often difficult to disambiguate wing flaps from postural adjustments associated with perch hopping. We excluded these measures (perch hopping and wing flaps) from further analysis, and the Results reported here are based on the expression of copulation solicitation displays or calls.

### Overview of Behavioral Testing

**Figure 1:**
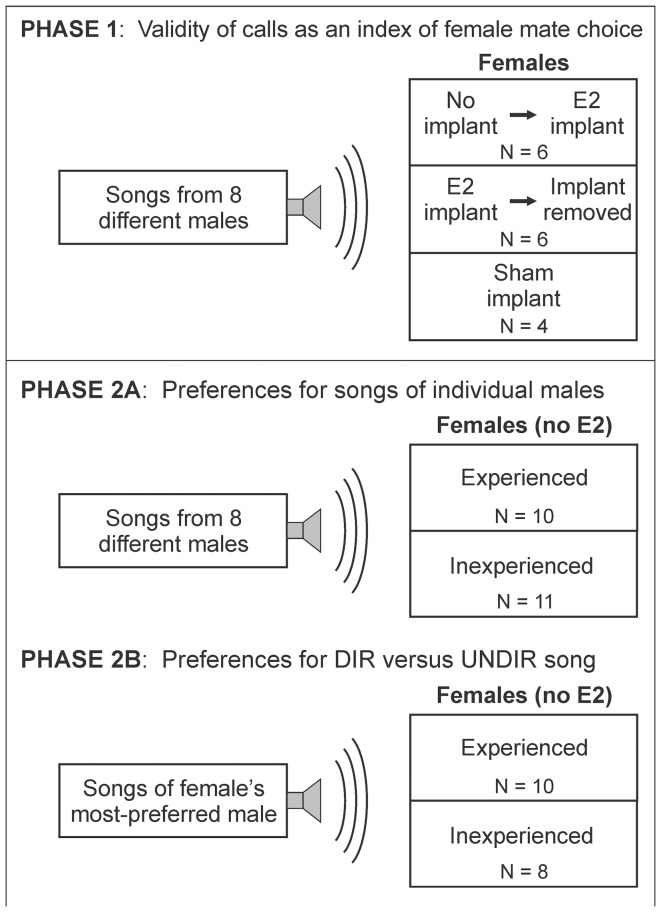
Overview of experimental methods and flow. In each Phase, we played song stimuli to female BFs in the absence of a male bird and tallied the number of responses to each stimulus. (A) In Phase 1, we investigated the degree to which calls as as reliable as CSDs as an index of female BF mate preference. In one group, females were tested first with no implant and then after a minimally invasive subcutaneous estradiol implant (N = 6 birds). In a second group, females were tested first with an estradiol implant and then after recovery from implant removal (N = 6 birds). In a third group, females were implanted with a subcutaneous sham implant (N = 4 birds). None of the birds tested in Phase 1 were also used in Phase 2. (B) In Phase 2A, we investigated the degree to which female BFs are consistent in their song preference across time and trials. In one group (“experienced”), females had interacted with the male birds from which song stimuli were recorded (N = 10 birds). In another group of birds (“inexperienced”), females had never interacted with those males (N = 11 birds). After a bird had completed Phase 2A, it moved on to Phase 2B in which we created stimuli from each female’s most-preferred male and tested the degree to which female birds prefer songs performed in the presence of a female (“directed”) versus songs performed when the male is alone (“undirected”). Three inexperienced birds failed to meet the criteria for inclusion in the results of Phase 2.

In one set of experiments (Phase 1, Figure 1), we defined the mate preference of each female BF by quantifying the expression of CSDs in response to playback of undirected song from each of 8 different BF males (N = 16 female birds). In those experiments (detailed in the following section), we also quantified the expression of calls during song playback to investigate the degree to which mate preference is evident in not only CSDs but also in expression of other forms of behavior. As detailed in the Results and as has been noted for another songbird species [33], we found that calls are as informative as CSDs in revealing female preference for specific songs. Therefore, we used calls to measure mate preference in all subsequent experiments.

In another set of experiments (Phase 2A, Figure 1), we investigated the degree to which female BFs express a preference for the song of one or more individual males and the degree to which that preference is consistent across time and repeated tests (N = 21 female birds that included none of the 16 birds used in tests of CSDs). In a final set of experiments (Phase 2B, Figure 1), we investigated the degree to which each female BF expresses a preference for directed song versus undirected song performed by her most-preferred male (N = 18 female birds that were a subset of the 21 birds used in Phase 2A). Methods through which preference was computed from the outcomes of tests in Phase 1, Phase 2A and Phase 2B are detailed at the end of this Methods section.

### Hormone Implantation Surgery and Tests of Copulation Solicitation Displays (Phase 1)

**Figure 2:**
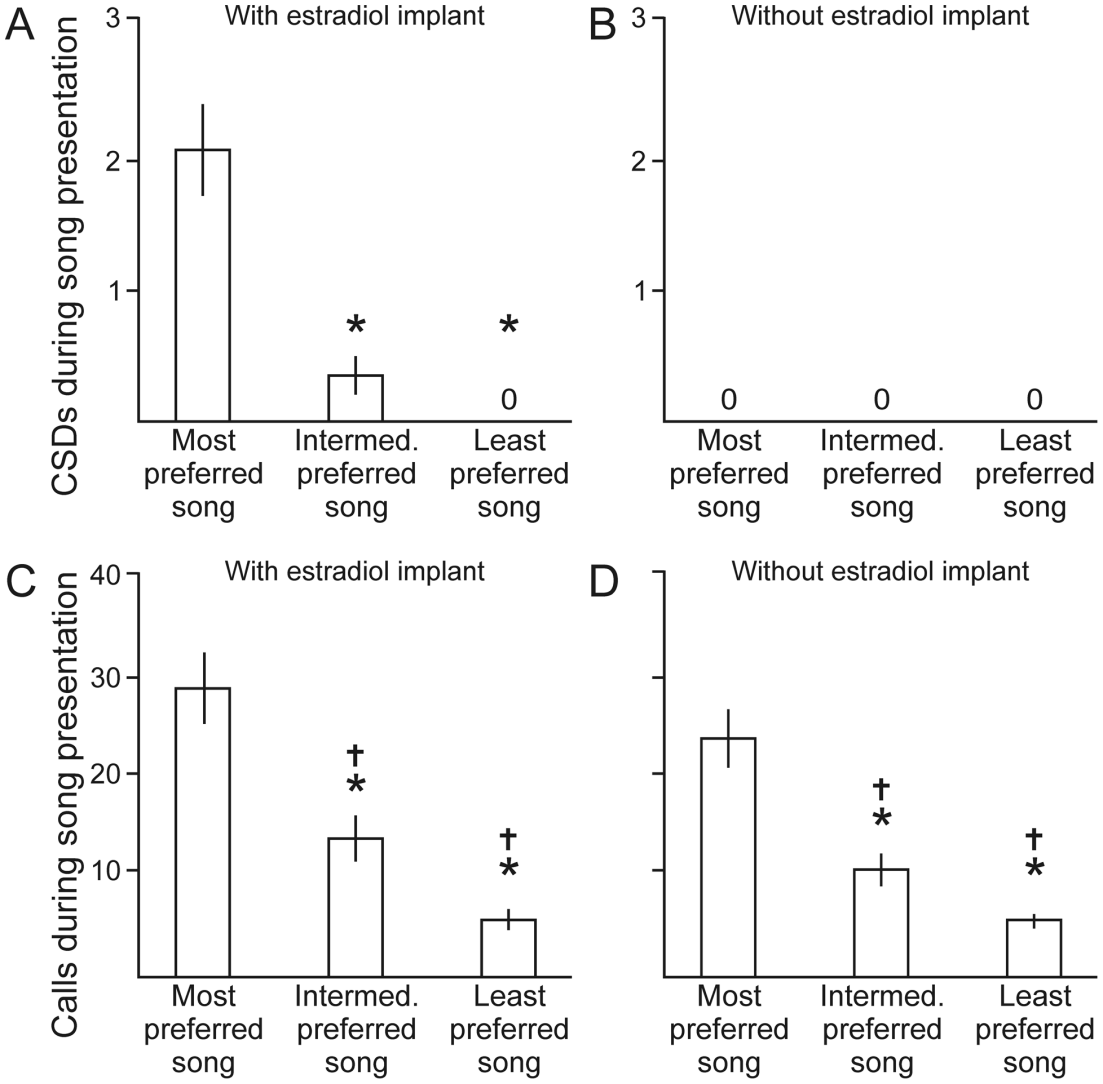
CSDs and calls are equally reliable measures of female BF mate preference. (A) With an estradiol implant administered, female BFs expressed CSDs in response to song playback, and song identity significantly affected that response. The most-preferred song evoked a significantly greater response than intermediately preferred songs or the least-preferred song (N = 12 birds; for all panels * indicates cases that are different than the response to the most-preferred song (Tukey’s HSD, p < 0.05); statistics are provided in the text unless otherwise noted here). (B) In the absence of an estradiol implant, birds never produced CSDs in response to any song stimulus (N = 12 birds). In no case did a sham implant induce birds to express CSDs in response to song (N = 4 birds, data not shown). (C) Calls produced during song presentation were also tallied in the same tests described in panels A and B. In the presence of an estradiol implant, birds called in response to many songs, and the song that evoked the greatest number of calls was invariably the same song that also evoked the greatest number of CSDs (intermediately-preferred songs and least-preferred songs evoked responses that were not only significantly different from the response to the most-preferred song (*, Tukey’s HSD, p < 0.05) but also different than one another (dagger symbol, Tukey’s HSD, q_s_ = 3.65, p = 0.04, N = 12 birds). (D) In the absence of an estradiol implant, birds also called in response to song stimuli (intermediately-preferred songs and least-preferred songs evoked responses that were not only different than the response to the most-preferred song (*, Tukey’s HSD, p < 0.05) but also different than one another (dagger symbol, Tukey’s HSD, q_s_ = 4.47, p = 0.01, N = 12 birds). The song that evoked the greatest number of calls without an implant was the same song that evoked the greatest number of calls in the presence of the estradiol implant in 11 of 12 birds. Therefore, calls are an excellent measure of mate preference in adult female Bengalese finches. More calls were produced in the estradiol condition than in the absence of estradiol, but that difference was not significant for any of responses to the most-preferred song (Mann-Whitney U test, p = 0.30, N = 12 birds) or intermediately-preferred songs (p = 0.17) or least-preferred songs (p = 0.91).

In the laboratory, female songbirds generally require a subcutaneous implant of estradiol in order to express CSDs commonly [2], [3], [6]. That was also the case in the BFs studied here (Figure 2A-2B), however other authors have reported varying results of estradiol in BF [5], [37] and have used other measures of female BF response to song [28]. We administered 17-ß-estradiol by subcutaneous implant in silastic tubing (1.96 mm outer diameter) containing 8 mm of hormone [6]. During the implantation procedure, the bird was restrained manually, a local anesthetic (4% lidocaine cream) was applied, the implant was inserted through a small incision in the skin overlying the abdomen, and the incision was sealed (VetBond). We monitored the bird throughout recovery. Seven days following that procedure, we presented female BFs with playback of the aggregate undirected song stimuli from the 8 males described above, and we recorded the expression of CSDs to define the mate choice of each female. We tested each bird’s preference 3 times in this paradigm.

Our female subjects expressed CSDs following estradiol implantation, but estradiol can affect auditory signal processing [38], and it remains unknown what effect that implant may have on the mechanisms of signal perception and evaluation over long periods. In an attempt to measure female BF mate choice over longitudinal studies in the absence of estradiol, we investigated the degree to which subjects expressed other behaviors in association with the performance of CSDs. We recorded our birds’ expression of calls and CSDs during song playback, and we tested birds in both the control and the hormone-implanted condition. In one set of subjects (N = 6), we tested each bird’s response first in the control condition and then again 7 days following estradiol implantation. In another set of subjects (N = 6, entirely distinct from the 6 birds in the previous group), we tested the birds first in the hormone-implanted condition and then again 6 weeks after removal of the implant to permit recovery from surgery and the effects of the implant. In a third set of birds (N = 4), we tested each bird’s preference following administration of a biologically inert sham implant (Kwik-Sil, World Precision Instruments) of similar size and shape as the hormone implant.

### Quantifying Preference for Song of an Individual Male (Phase 2A)

In the tests of Phase 2A, we presented females with the aggregate undirected song stimulus for each of 8 males (the same stimuli used in Phase 1), and we recorded responses to quantify each female’s preference for songs of different individuals. We tested each bird’s preference 5 times in this paradigm. Numbers of responses could vary considerably between birds and even between tests of the same bird. To ensure that each test included sufficient data that a preference could be determined, we enforced the criterion that a valid test of preference was one in which the female called at least 10 times in response to the song of one individual male or at least 4 times each to the songs of at least 2 different males. If a bird failed to reach 5 valid tests by the time that it had been tested 10 times, then testing for that bird was discontinued and it was excluded from these results (all birds responded with at least one call to one song stimulus, but 9 birds did not meet this criterion and were excluded). We considered females to be valid if they reached 5 valid tests before that point (21 birds passed). Thus, in Phase 2A we quantified the number of calls that each female produced in response to each male bird and used those data to identify the most-preferred male for each female.

Calls produced by our birds were multi-note trills of the sort described previously as amplitude modulated calls in adult female Bengalese finches [39]. The number of trilled notes in each call ranged from 2 to 4, and each bird produced each type of trill at least once in response to the most-preferred song. Our initial analyses revealed no difference between the length of trills (number of trilled notes) that each female produced in response to her most-preferred song versus other songs (One-way ANOVA, F(7,72)  = 1.54, p = 0.17, N = 10 birds). Furthermore, calls were also commonly accompanied by vigorous activity including approaching the side of the cage nearest the speaker. Because of the dimensions of the testing apparatus, it was difficult to assess the relation between approach and preference, but approaching the source of the sound was commonly observed in association with songs that evoked large numbers of calls. We tallied the total number of calls that each female performed in response to all undirected song stimuli and used that number to calculate a selectivity index for each bird. This index is the magnitude of the female’s response to her most-preferred male divided by the average response across all males. This value is 1 for a female that is equally responsive to all males and much larger for birds that are strongly selective for the song of an individual male(s).

### Quantifying Preference for Directed Song (Phase 2B)

After a bird had completed Phase 2A, we transitioned the female into Phase 2B. That phase consisted of tests designed to measure whether the bird expressed a preference for directed or undirected variants of the song of her most-preferred male. We presented each female with the aggregate undirected song stimulus (approximately 60 sec) and the aggregate directed song stimulus (approximately 60 sec) of her most preferred male, and we recorded responses to quantify each female’s preference for either of those song variants. We tested each bird’s preference 3 times in this paradigm, and we enforced the same criteria for a valid test that we enforced in Phase 2A. We excluded birds from these results if they failed to reach 3 valid tests by the time that we had tested them 6 times (3 birds excluded from the set of 21 birds that passed Phase 2A). To quantify each female’s selectivity for directed versus undirected song, we tallied the total number of calls that each female performed in presentations of undirected and directed variants of the song of her most-preferred male, and we used that to compute the female’s preference for either variant, which we quantified as the percent response to directed song minus the percent response to undirected song. Using this metric, positive values indicate a greater response directed song, and negative values indicate a greater response to undirected song.

### Considering the Possible Influence of Each Female’s Experience with the Male Singers Used to Test Female Song Preference

In each of the experiments in Phases 2A and 2B, we used two separate cohorts of female birds to begin to examine the role of experience in female BF preference for male song. The first group contained females that were alive at the same time as the males that we used to test female preference (N = 10 birds). To our knowledge, none of the females tested in that group had ever mated with any of the males used to test preference, however because of that contemporary relationship, it is likely that those males and females interacted. To control for that possible influence of experience (e.g., auditory, visual, social), we tested a second group of females that all hatched after all of the male singers were deceased (N = 11 birds; testing for the second group began 14 months after the start of testing for the first group). Therefore, the members of the first and second cohort had grossly similar experiences living in the colony, but the members of the second cohort were naïve to the songs of the males used in this study. All handling and testing procedures were identical between the two groups.

### Quantifying Features of Male Songs

We computed the following measures for each of the undirected and directed songs of each of the 8 males we used in tests of female preference (values were computed using custom Matlab software unless otherwise noted). *Tempo (notes per sec):* We computed tempo by dividing the total number of notes by the total duration of each stimulus. *Song duration (sec):* This is the duration of the entire song, including all epochs of sound and silence. *Maximum frequency (Hz), mean frequency (Hz), minimum frequency (Hz), spectral entropy:* We computed these parameters using Sound Analysis Pro software [40]. The frequency values were the maximum, minimum, and mean of all sounds present in the complete song stimulus. Spectral entropy (Weiner entropy) is a measure of the width and uniformity of the power spectrum, and we computed this value using algorithms detailed in Sound Analysis Pro and in publications describing that software [40]. Entropy ranges from 0 for complete disorder to progressively more negative values for progressively more ordered structure, with negative infinity representing a completely ordered signal. *Number of unique note types in each song:* This is the number of distinct note types present in each song. *Sequence transition entropy:* We computed this value using the methods described in Sakata et al 2008 [31]. A value of 0 indicates that the syllable sequence is completely fixed. Songs containing progressively more variable syllable sequences have higher transition entropies. *Sequence linearity, sequence consistency, sequence stereotypy:* We computed these values using the methods of Scharff and Nottebohm 1991 [41]. Sequence linearity provides a measure of how song notes are ordered, with a value of 1 for a completely stereotyped order and a positive number approaching zero for a highly variable note sequence. Sequence consistency addresses how often a particular note sequence, the most common note sequence in that song, is performed. A value of 1 indicates a completely consistent song in which all note transitions are part of that most common sequence, and lesser consistency is indicated by a smaller value approaching zero for a highly variable note sequence. Sequence stereotypy is computed using the values of sequence linearity and sequence consistency. Sequence stereotypy provides an easily interpretable summary of sequence features, as its value ranges from 1 for a completely stereotyped, highly ordered song to numbers approaching 0 for songs containing highly variable note sequences. *Number of epochs of note repetition in each song, number of note repeats in each epoch of repetition, percent of song duration comprising repetition:* We computed these values using custom software (Matlab). They represent the number of epochs in each song when any note was repeated, the number of times that the note was repeated in each of those epochs, and the total number of note repeats in each song divided by the total number of notes in the song, respectively. We used these parameters to compare each female’s most-preferred song versus other songs in the stimulus set and to compare undirected versus directed variants of each male’s song.

### Statistical Analyses

We used appropriate parametric or nonparametric statistical tests to compare results obtained under different conditions, as reported in association with the results of each comparison. Briefly, in Phase 1 we used a two-way ANOVA to compare the number of responses to each of 8 different song stimuli and in 2 different sequences of estradiol implantation (song was one factor with 8 levels, and testing sequence (either estradiol control, or control estradiol) was another factor with 2 levels). Post hoc tests were used to investigate possible differences in the responses to different song types. As elaborated in the Results, those data clearly indicated that the most-preferred song evoked a different response than other song types. To simplify our comparison of the most-preferred song type versus other degrees of preference, we used the number of CSDs performed by each bird in the estradiol-implanted condition to identify three stimulus categories for each bird: 1) the “most-preferred” song that evoked the greatest number of CSDs in that bird, 2) the “least-preferred” song that evoked the fewest CSDs in that bird, and 3) an “intermediately-preferred” song type. The methods through which we identified the songs that composed each category are as follows. In the case of the most-preferred song for each bird, that unique distinction was always evident in the behavioral response data, as there was never a tie between the number of CSDs evoked by the 1^st^ and 2^nd^ ranked song types. In the case of the least-preferred song type for each bird, it was common that multiple songs would be associated with expression of zero CSDs. In that case, we randomly selected one of those song types as the least-preferred song type. In the case of the intermediately-preferred song type for each bird, we randomly selected a song type from among the remaining 6 stimuli that were neither the most-preferred nor the least-preferred song type. These stimulus categories, which were defined using CSD data in the estradiol-implanted condition, were maintained when we considered the number of calls that each bird produced in response to each song. As detailed in the Results, the song that evoked the greatest number of CSDs invariably also evoked the greatest number of calls. Therefore, the term “most-preferred song” refers to the song that evoked the most CSDs in each bird, and that term also refers to the song that evoked the greatest number of call responses in that same bird.

In Phase 2A, we used a one-way ANOVA and post hoc tests to compare the number of calls that each bird produced in response to songs of 8 different males. In Phase 2B, we used chi-squared tests to compare the number of calls that those same birds produced in response to directed and undirected variants of the song of each female’s most-preferred male. In tests of the degree to which song preference is related to experience with the associated singers, we used a Kolmogorov-Smirnov 2-sample test to compare the results obtained from females that were alive at the same time as the males whose songs were used to test preference versus results obtained from birds that were not alive at that time. Finally, we used a one-way ANOVA and post hoc tests to compare the properties of each of the 8 song stimuli used in Phase 2A and each of the directed versus undirected song variants used in Phase 2B (separate tests for each song property). We used the Mann Whitney U test in other comparisons of two quantities in which the data are not parametrically distributed. We express each of our results as mean ± SE unless otherwise noted.

## Results

### Calls during song presentation are an indicator of female sexual preference

In Phase 1, we presented female BFs with playback of song from 8 different BF males and counted the number of copulation solicitation displays performed in response to each song following a subcutaneous implant of estradiol (N = 12 birds). For each bird, there was a “most-preferred song” for which more CSDs were performed than in response to the song of any other bird, but many songs evoked no CSDs at all (identity of the most-preferred song varied across females, considered again in later sections). Song had a significant effect on CSD expression in the estradiol-implanted condition (Two-way ANOVA, F(7,95)  = 53.51, p < 0.001, N = 12 birds), but the sequence of testing (control estradiol or estradiol implant removed) had no effect on CSD expression (F(1,95)  = 0.63, p = 0.43, N = 12 birds) and there was no significant interaction (F(1,7)  = 0.31, p = 0.95, N = 12 birds). Females expressed significantly more CSDs in response to the most-preferred song than in response to any other stimulus (Tukey’s HSD, q_s_ > 18.01 and p < 0.05 for all 7 comparisons of most-preferred song type versus all other song types, N = 12 birds).

To simplify our comparison of responses to the most-preferred song type versus responses to stimuli with lesser subjective value, we also identified the least-preferred song type and an intermediately preferred song type for each bird (details in Methods). As in the previous result, analysis using this categorization of stimuli revealed that song had a significant effect on CSD expression (Figure 2A, Two-way ANOVA, F(2,35)  = 52.12, p = < 0.001, N = 12 birds), the sequence of testing had no effect (F(1,35)  = 0.30, p = 0.59, N = 12 birds), there was no significant interaction (F(1,2)  = 0.30, p = 0.74, N = 12 birds), and females expressed significantly more CSDs in response to the most-preferred song than in response to the least-preferred or intermediately-preferred stimuli (Tukey’s HSD, q_s_ > 11.44 and p < 0.05 for both comparisons, N = 12 birds). Therefore, the most-preferred song is easily identified by observing CSDs, and next we investigated whether that preference was also evident in the expression of calls.

We recorded our estradiol-implanted birds’ expression of calls during song playback and found that song identity also significantly influenced calling behavior (Two-way ANOVA, F(7,95)  = 39.53, p < 0.001, N = 12 birds) but the sequence of testing had no effect (F(1,95)  = 2.48, p = 0.12, N = 12 birds) and there was no significant interaction (F(1,7)  = 1.10, p = 0.37, N = 12 birds). As in the case of CSDs, that influence on calls was also evident even when we categorized our stimuli. Specifically, song identity influenced calling behavior (Figure 2C, Two-way ANOVA, F(2,35)  = 51.83, p < 0.001, N = 12 birds), the sequence of testing had no effect (F(1,35)  = 0.84, p = 0.37, N = 12 birds), there was no significant interaction (F(1,2)  = 1.99 p = 0.15, N = 12 birds), and females called significantly more in response to the most-preferred song than in response to any other stimulus (Figure 2C, Tukey’s HSD, q_s_ > 3.65 and p < 0.05 for both comparisons of most-preferred song type versus other song types, N = 12 birds).

**Figure 3:**
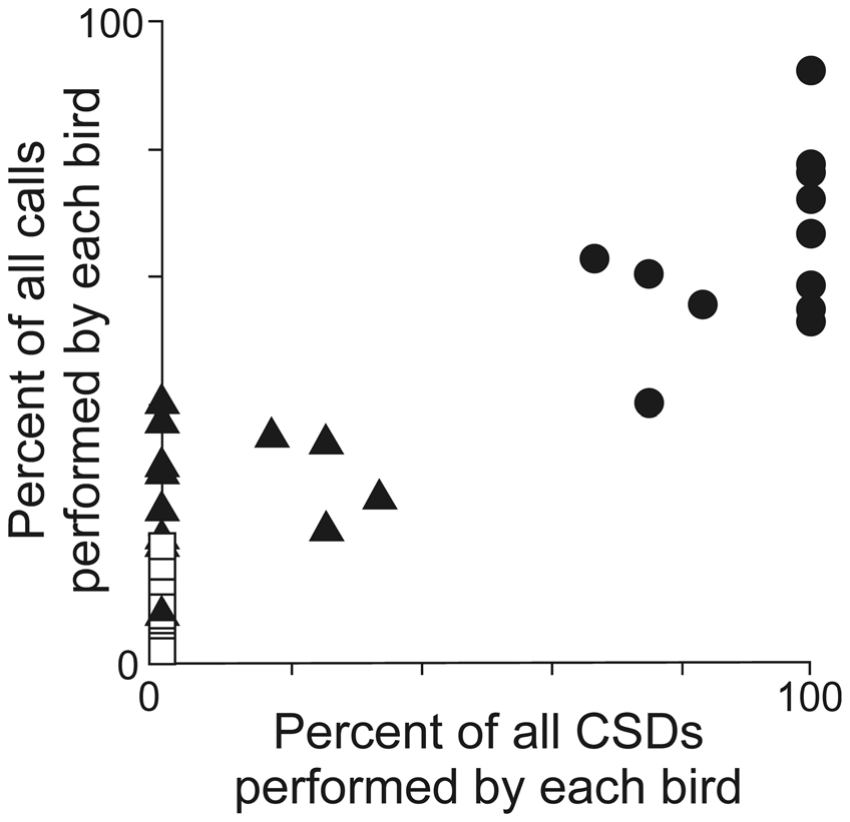
Calls are an index of female BF mate choice. Each female is represented by 3 data points (N = 12 birds, some points overlap) indicating the magnitude of its response to its most-preferred song (filled circles), its least-preferred song (open squares), and a randomly selected song of intermediate subjective value (filled triangles). Each of the three responses from each bird have been normalized to the total number of responses performed by that bird in response to all stimuli, so data are plotted as the percent of all CSDs (x-axis) and all calls (y-axis) that each bird performed in response to all stimuli. These data reveal good agreement between CSDs and calls (statistics detailed in the text), indicating that calls are a valid index of adult female BF mate choice.

When the same birds were tested in the absence of an estradiol implant, the birds did not express CSDs (Figure 2B), but song identity had a significant effect on calling (Figure 2D, Two-way ANOVA, F(2,35)  = 76.45 p < 0.001, N = 12 birds), the sequence of testing had no effect (F(1,35)  = 2.29, p = 0.14, N = 12 birds), and there was no significant interaction (F(1,2)  = 2.92, p = 0.07, N = 12 birds). Even without an estradiol implant, females called significantly more in response to the most-preferred song than in response to any other stimulus (Figure 2D, Tukey’s HSD, q_s_ > 4.47 and p < 0.05 for both comparisons of most-preferred song type versus other song types, N = 12 birds). Therefore, the most-preferred song is also easily identified by counting the number of calls that the female produces during song playback. To further investigate the relation between CSDs and calls, we compared the percentage of each bird’s total number of CSDs versus the percentage of each bird’s total number of calls that were evoked by each of the most-preferred, least-preferred and intermediately-preferred song types, and we found good agreement between these measures (Figure 3, Spearman rank correlation coefficient, r_s_ = 0.82, p < 0.01, N = 12 birds). Together, these data establish a link between mate choice and the expression of calls in response to song presentation. In the remaining experiments, we used calls to quantify each female bird’s mate preference.

### Mate preferences of individual females are consistent across time and tests

**Figure 4:**
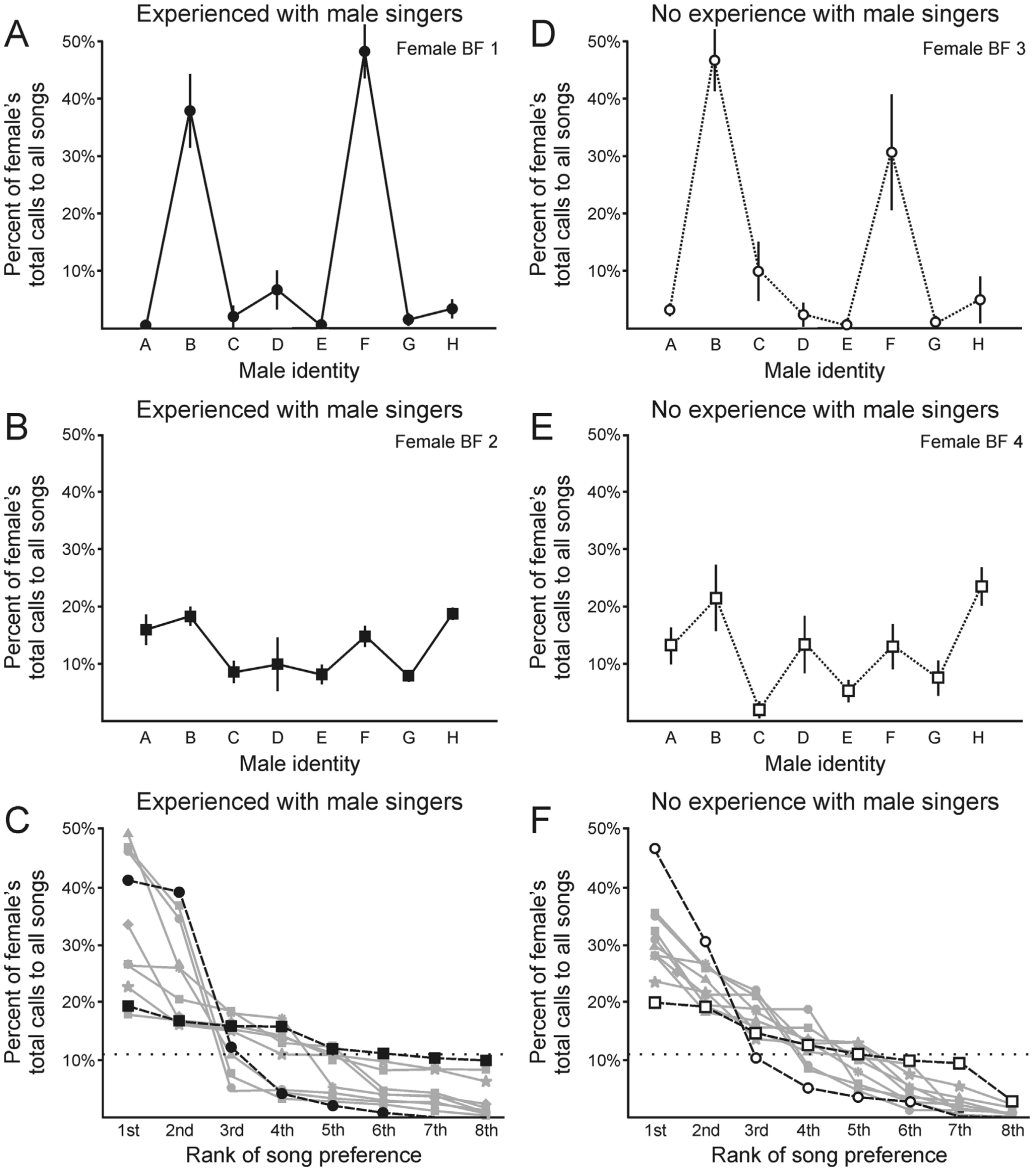
Individual females vary in their selectivity for songs of different males. Individual females expressed a range of selectivity for the songs of individual males, and the nature of those preferences was not different between birds that did (panels A-C) or did not (panels D-F) have previous experience with the associated male singers. (A) Among birds with experience of the male singers, some females were very selective for the song(s) of individual males, such as the female shown here (selectivity index  =  3.76; points indicate mean; lines indicate SE in panels A, B, D, E). (B) Other females were not selective, responding similarly to the songs of many males, as in the case of the female shown here (selectivity index  =  1.43). (C) Across all 11 birds that had experience with the male singers, selectivity indices ranged from 1.43 to 3.97 (mean ± SE  =  2.65±0.31, dark solid lines indicate the birds shown in panels A (filled circles) and B (filled squares), dotted line indicates level of chance). (D) Among birds that did not have experience with the male singers, the response were very similar, with one bird expressing very selective responses (selectivity index  =  3.76) and (E) another bird responding much more broadly (selectivity index  =  1.89). (F) Across all 10 birds that did not have experience with the male singers, selectivity indices ranged from 1.60 to 3.76 (mean ± SE  =  2.47±0.17) and were indistinguishable from those detected for birds that had experience with the singers (statistics detailed in the text; dark solid lines indicate the birds shown in panels D (open circles) and E (open squares), dotted line indicates level of chance).

To characterize mate preference for each bird, we tested each female’s response to a suite of songs from each of 8 BF males. In this Phase 2A of testing, the preference of each female for each of the 8 males was quantified over the course of 5 tests for each bird (e.g., Figure 4A). The pattern of song preference that characterized each female was stable across time and tests spanning intervals as short as 3 hours and as long as 130 days (interval between tests: 13.22±2.79 days, N = 21 birds). For example, the bird described in Figure 4A expressed a rather selective preference for the songs of males B and F, and the bird described in Figure 4B was broadly responsive to the songs of many males. Although the identity of the most-preferred male differed between individual females (detailed below), each bird’s preference was consistent across time and tests, as evident in the small standard error bars in Figures 4A-4B and 4D-4E (across all 10 birds standard errors were also quite small: 3.0±0.4% on the scale used in Figures 4A-4B and 4D-4E, corresponding to a coefficient of variation of 0.35±0.03). Consistency in the preference of each individual female indicates that female BFs are capable of recognizing conspecific males by their songs, and female preferences are systematically related to some feature or suite of features that characterize each song.

### Mate preferences vary across different females

**Variability in selectivity index.** Some female BFs were quite selective in their mate preference, as revealed by strong responses to the song of only one or two males and little or no response to the songs of other males (e.g., Figure 4A, selectivity index  = 3.76). Other females were much less selective in their mate preference, as revealed by similar responses to the songs of many or all males (e.g., Figure 4B, selectivity index  =  1.43). Together with the consistency of individual preferences across time and tests (detailed above), these differences between birds indicate that each female BF expresses a characteristic pattern of mate preference, with each female typically expressing a strong preference for the song of only one or two males (all 10 birds in this dataset are summarized in Figure 4C, mean selectivity index  =  2.65±0.31).

**Mate preference is directed by song rather than interaction with the associated singer.** The finding that individual females typically prefer the song of only one or two males suggests that female BF mate preference is related to some acoustic feature or set of features that distinguish those songs. However, another possibility is that females use the song to identify their male of choice [35], but their preference is related to some other aspect of experience with that male. For example, the females that were tested in the dataset described in Figures 4A-4C were all alive at the same time as the males from which we recorded the songs used to test mate preference (“experienced group”, N = 10 birds). Therefore, it is possible that those females had encountered those males in the colony, identified specific males by their song, but evaluated the quality of those potential suitors based on other traits such as plumage or social hierarchy. To account for that possibility, we repeated the Phase 2A tests described in Figures 4A-4C, however in this second iteration we performed our tests using adult female BFs that had never interacted with any of the males from which we recorded songs used to test mate preference (“inexperienced group”, N = 11 birds, Figures 4D-4F). Therefore, there was no possibility that those inexperienced females had any sensory or social experience with the males used in tests of song preference. As in the case of experienced females, these tests using inexperienced females revealed that some females were selective, with strong responses to song of only one or two males (e.g., Figure 4D, selectivity index  =  3.76), and other inexperienced females were much less selective (e.g., Figure 4E, selectivity index  =  1.60). Preferences were generally very similar between experienced and inexperienced females. Specifically, no difference in selectivity index could be detected between experienced females (Figures 4A-4C) and inexperienced females (Figures 4D-4F, Mann-Whitney U test, p = 0.61; a power test reveals that 81 birds would have been necessary to have statistical power of 0.8). Although this does not exclude the possibility that a subtle difference may exist in the selectivity of these two populations, the general similarity of these values suggests that experience with the singers of specific song stimuli has little or no effect on a female’s selectivity for those stimuli. Individual-specific mate preferences were also consistent for inexperienced females (standard error bars as shown in Figures 4A-4B and 4D-4E were of magnitude 5.0 + 0.4%, corresponding to a coefficient of variation of 0.42±0.03). Other features of female mate preference were also similar between the experienced and the inexperienced groups, as described in the following text and figures in which different symbols or different panels are used to distinguish between females with or without experience of the associated male singers.

**Figure 5:**
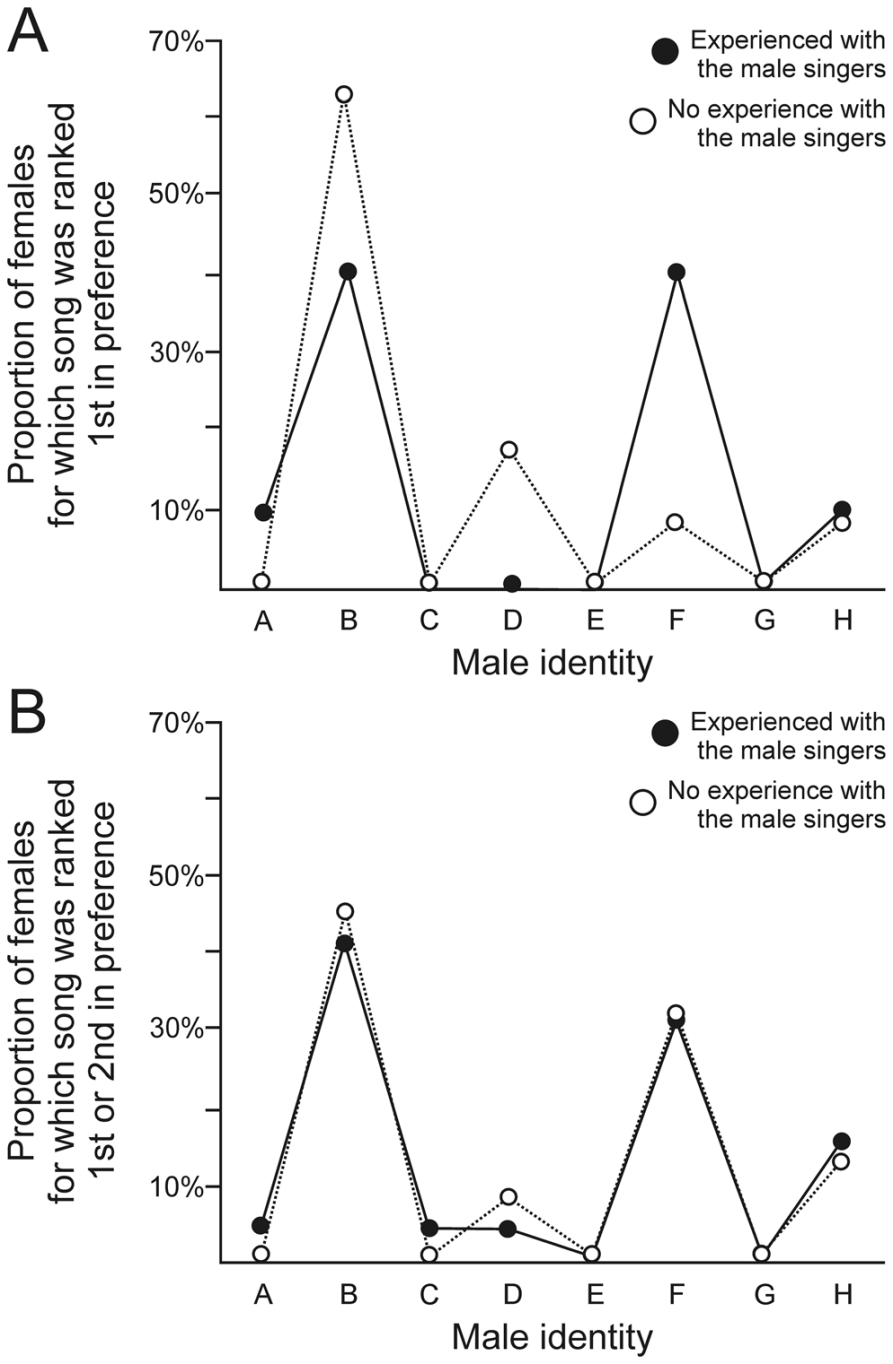
Females prefer songs of some males more than others. Females tended to prefer the songs of two males more than the other males tested here, and preferences for individual males were broadly similar between birds that did or did not have previous experience with the associated male singers. (A) Each female had one song for which she generated more calls than in response to any other song, which was deemed the most-preferred song type for that bird. Across all 21 birds tested, songs of two males (identified here as B and F) were more commonly preferred than the songs of other birds (filled symbols  =  10 females that had experience with the associated singers, open symbols  =  11 females that did not have experience with the associated singers). (B) When we expanded that consideration to include not just the top-ranked song for each bird but also the second-ranked song for each bird, that broad preference for males B and F was also evident (symbols as in panel A). Across the 21 birds tested here, 5 different songs were ranked as the most-preferred stimulus, and 6 different songs were ranked as either the top-ranked song or the second-ranked song. Across the population (N = 21 birds), male identity had a profound effect on female preference, but population preferences were similar regardless of whether the females did or did not have experience with the male singers (statistics detailed in the text).

**Variability in identity of the most-preferred male.** Across the population of females tested here, song identity had a strong influence on female mate choice (Figure 5A, One-way ANOVA, F(7,168)  = 80.86, p < 0.001, N = 21 birds). There was also considerable variability in the identity of the male that each female identified as her most-preferred male. Specifically, across the 21 females tested here, there were 5 songs that were ranked as at least one female’s most-preferred song (Figure 5A). To more broadly describe the preferences detected across the population, we also identified not only each female’s most-preferred song (first ranked in Figures 4C or 4F, summarized in Figure 5A) but also the song that each female identified as second most-preferred. When the dataset was analyzed with that more inclusive perspective, we identified 6 songs that were ranked as either most-preferred or second most-preferred by at least one of the 21 females. Specifically, the males designated B and F in Figure 4 were very commonly preferred, with 6 of the 21 females (29%) ranking one of those males as their most-preferred song (Figure 5B).

Importantly, the tendency for females to prefer the songs of those two males was evident both in the population that had experience with the associated male singers and the population that had no experience with those males. This broad similarity in the mate preferences of these two groups (Figure 5B; distributions were indistinguishable between groups, Kolmogorov-Smirnov two-sample comparison, p = 0.93, N = 8 songs) reveals that female preference for the song of a specific male does not require sensory or social experience with that male. Instead, female preference is strongly directed by acoustic features of the song itself. Therefore, our data reveal that BF females express a range of individual-specific preferences for specific song features, yet there are nonetheless some songs that contain features of sufficiently broadly relevant attractiveness that they evoke at least some courtship behavior from a range of different females.

To begin to investigate what features may be most important in distinguishing those broadly attractive songs from the songs of other less attractive males, we quantified a spectrum of properties for the undirected songs of each of the 8 males used in Phase 2A tests of female preference. We found that for only 6 of the 14 properties that we measured, the song of either male B or male F was superlative (had either the greatest of the least magnitude) among the songs of all 8 males. Those 6 properties included mean tempo, mean frequency, spectral entropy, the number of epochs of note repetition, the number of repeated notes in each epoch, and the percent of song duration that comprised note repetition. We further investigated that relation by computing the correlation between the magnitude of those values for each song and the percent of all calls that each song evoked. In only 3 cases was that correlation coefficient (Spearman r_s_) significant (p < 0.05) and greater than 0.50 (mean tempo, mean number of repeated notes, percent of song duration comprising note repetition). These correlations suggest that song tempo, the extent of repeated notes in the song, or some combination of those features may be important in female evaluation of mate quality. Defining the role of these features together or alone in shaping female preference will require additional experimentation in future studies.

**Figure 6:**
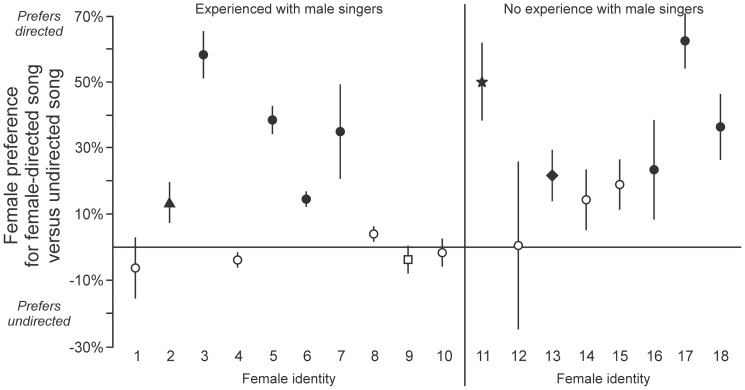
Females commonly prefer directed songs more than undirected songs. Among the birds that expressed a significant preference in tests of directed versus undirected song, all preferred directed song. Among birds that had experience with the male singers (left, birds 1-10), 5 expressed a significant preference for directed song (chi-squared test, p < 0.05, filled symbols) and 5 expressed no significant preference (p ≥ 0.05, open symbols) (square indicates bird in Figure 4A, triangle indicates bird in Figure 4B). Among birds that did not have experience with the male singers (right, birds 11-18), 5 birds expressed a significant preference for directed song and 3 birds expressed no significant preference (open and filled symbols as in birds 1-10; star indicates birds in Figure 4D, diamond indicates bird in Figure 4E). Preference for directed song was evident across different males but was ultimately specific to each female, as the 10 significant responses (filled symbols) correspond to 5 different males, but not all females that heard those songs preferred directed song.

**Variability in preference for female-directed song.** Previous studies of other songbird species indicate that females prefer songs that males perform in the company of a female and direct specifically to that female receiver (“directed” songs) more than songs that males perform when they are alone (“undirected” songs) [32]. To investigate that possibility for the BFs studied here, we performed Phase 2B tests of each female’s preference for directed versus undirected songs performed by her most-preferred male. Some females expressed a significant preference for directed song (filled symbols in Figure 6, p < 0.05 in chi-squared tests), other birds expressed no significant preference (open symbols in Figure 6), but no female expressed a significant preference for undirected song over directed song. Thus, among those birds that did express a significant preference, all of them preferred directed song over undirected song (N = 18 birds). That tendency was evident both in the group of experienced females (Figure 6, birds 1-10) and in inexperienced females (Figure 6, birds 11-18). Consistent with results from other species [32], our behavioral data reveal that the properties of directed songs are sufficiently different from those of undirected songs that females can recognize them as distinct. Our data also reveal that the differences between directed and undirected songs are relevant to signal evaluation and mate selection. In many cases, those changes cause the song to become more attractive to the female receiver. In other cases, there is little or no effect on the female’s response, however in no case do those changes cause the song to become less attractive than the song performed when the male is alone.

Following on our investigation of the properties that distinguished the songs of specific males in Phase 2A, we also quantified a spectrum of properties for the undirected and directed songs of each of those males (Table 1; undirected and directed songs were analyzed from each of the 8 male birds that were the source of the song stimuli used in Phases 1 and 2A of testing). In no case were any of the properties consistently increased or decreased in the directed songs of each male. An especially informative case may be the song of the male that was most broadly preferred among all the females tested and was therefore used most commonly in tests of preference for directed versus undirected song (male B in Figures 4 and 5). For that male, the largest changes between the undirected and directed states (changes of at least 10% of the value in the undirected state) were evident in mean tempo, sequence linearity, sequence stereotypy, the number of epochs of note repetition, and the percent of song duration comprising note repetition (Table 1). Taken together, these changes reveal that the directed song of that male had a faster tempo, was more variable in its sequence, and contained less note repetition than the undirected song of the same bird. These findings leave open the important question of what song features are most salient in directing female mate preference, but they highlight several candidate features and the degree to which they may be positively or negatively correlated with female preference.

**Table 1:** Parameters of Directed and Undirected Songs of Each Male Used in these Experiments

	Male Identity															
	Male A		Male B		Male C		Male D		Male E		Male F		Male G		Male H	
	UNDIR.	DIR.	UNDIR.	DIR.	UNDIR.	DIR.	UNDIR.	DIR.	UNDIR.	DIR.	UNDIR.	DIR.	UNDIR.	DIR.	UNDIR.	DIR.
Tempo (notes/s)	8.74	8.42	12.93	15.48	8.61	7.70	9.52	8.79	7.15	8.76	9.18	10.99	7.86	11.00	6.66	6.41
Song duration (s)	74	77	56	58	77	75	55	54	56	54	59	60	46	47	65	68
Max. frequency (Hz)	10248	8870	9042	8784	9732	10248	10076	8956	12660	9560	10680	10594	11024	10852	10852	10680
Min. frequency (Hz)	806	806	978	892	892	892	892	806	978	906	1150	1064	806	806	822	1064
Mean frequency (Hz)	3384	3472	4138	4263	3800	3436	3836	3934	3884	3788	3793	3678	3618	4085	3909	3843
N different note types	10	10	7	7	6	6	6	6	7	7	7	7	9	8	6	6
Spectral entropy	-2.61	-3.06	-2.87	-3.11	-2.53	-2.66	-2.35	-2.51	-2.18	-2.04	-2.95	-2.87	-2.74	-2.16	-2.02	-2.05
Sequence entropy	4.41	4.13	2.85	2.93	3.88	3.68	2.91	2.33	2.34	2.88	3.47	3.64	4.33	4.01	2.58	2.79
Sequence linearity	0.56	0.58	0.47	0.30	0.42	0.27	0.54	0.41	0.50	0.30	0.37	0.25	0.50	0.24	0.49	0.35
Sequence consistency	0.74	0.76	0.74	0.71	0.63	0.58	0.70	0.75	0.70	0.65	0.59	0.56	0.80	0.59	0.74	0.72
Sequence stereotypy	0.65	0.67	0.60	0.51	0.52	0.43	0.62	0.58	0.60	0.47	0.48	0.40	0.65	0.41	0.62	0.54
Number of epochs of note repetition	12	12	12	10	6	7	20	13	8	6	23	16	3	3	7	7
Average number of repeated notes in each epoch	2.38	2.35	3.50	3.49	1.00	1.15	1.41	1.40	1.44	1.30	1.84	1.76	1.25	1.06	1.75	2.03
Percent of song duration comprising note repetition	35.0	28.4	41.5	35.6	14.4	13.8	51.7	29.5	17.3	10.2	51.9	47.4	8.4	9.3	18.7	19.8

### Preferences for different aspects of male song are independent

To begin to understand how perception of song quality may be encoded in the female brain, we investigated the degree to which specific song features are evaluated independently. In other words, we investigated whether a female that was choosy for the identity of an individual singer was similarly choosy for directed versus undirected song. Similar degrees of choosiness for multiple song features would suggest that perception of those different features is accomplished through activation of one and the same neuronal population or separate populations that are strongly linked. In contrast, no relation between the degree of choosiness for different features would suggest that perception of those features is accomplished through activation of separate populations of auditory processing neurons. Thus, an analysis of the degree to which different facets of female preference are correlated with one another will enable us to formulate hypotheses about the neural circuitry through which song-based mate preference emerges.

**Figure 7:**
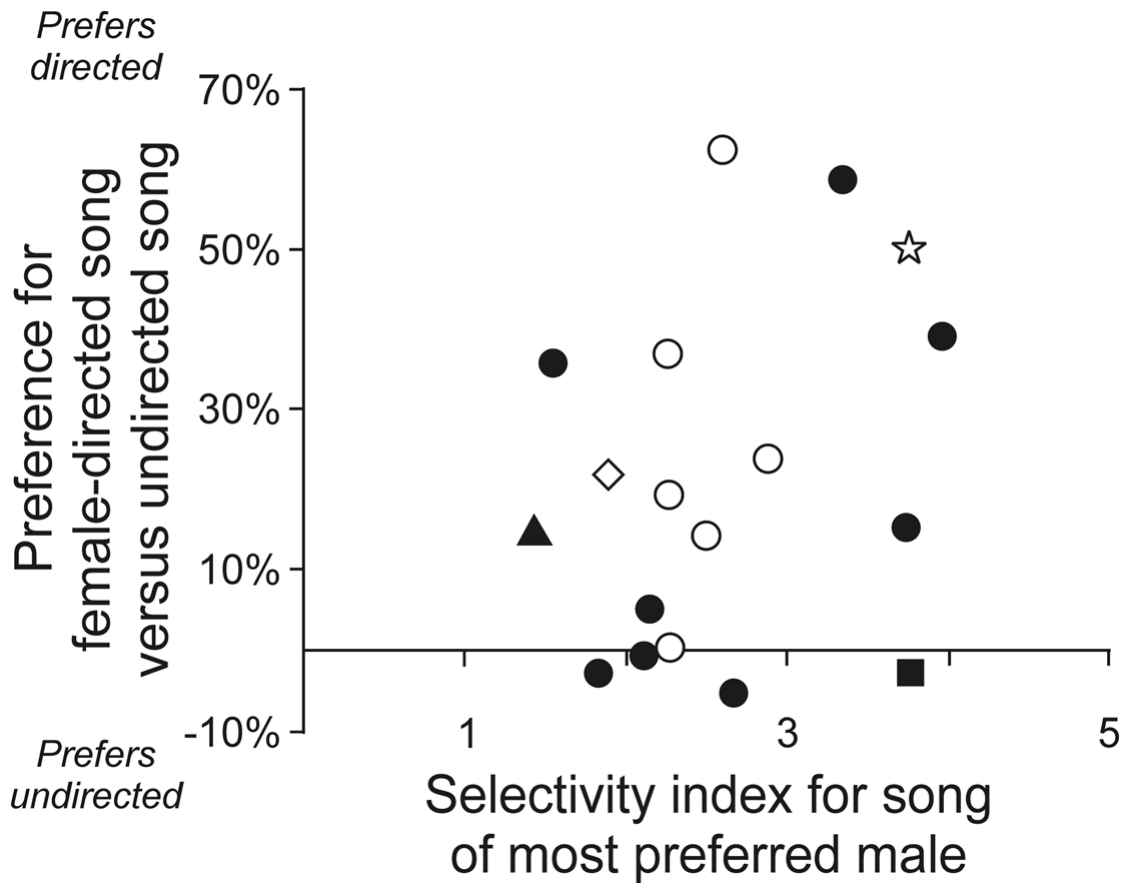
Different facets of male song can be evaluated independently. When different facets of song selectivity are compared for each bird, the degree of selectivity for individual identity and the degree of selectivity for directed song are not related. That was true for both the group that had experience with the male singers (filled symbols; square indicates bird in Figure 4A, triangle indicates bird in Figure 4B) and females that had no experience with the male singers (open symbols; star indicates birds in Figure 4D, diamond indicates bird in Figure 4E; statistics detailed in the text). Thus, an individual bird can be selective for one aspect of male song but unselective for another aspect.

We quantified each female’s selectivity for the songs of different males (selectivity index, Figure 4) and compared that to her selectivity for directed song (Figure 6). Across the population of female birds, there was no significant relation between those variables (Figure 7), evident as a slope that was not different than zero (Linear regression, p = 0.27, regression  =  18 birds) and no significant correlation (Spearman correlation, p = 0.28, N = 18 birds). The absence of a relation between selectivity for individual identity and selectivity for directed versus undirected song was evident in birds that had experience with the male singers (Linear regression slope not different than zero: p = 0.72, Spearman correlation not significant, p = 0.67, N = 10 birds, filled symbols in Figure 7) and in birds that had no experience with the male singers (Linear regression slope not different than zero: p = 0.21, Spearman correlation not significant, p = 0.35, N = 8 birds, open symbols in Figure 7). These data reveal that selectivity for one facet of male song does not obligate a female to be equally selective for other facets of that song. Therefore, preference is not a monolithic entity. Instead, female BF mate choice is influenced by multiple facets of male song performance, and at least two of those facets are evaluated independently. Evidence of independent evaluation of specific song features gives rise to the very interesting possibility that evaluation of different song features may be independent cognitive processes associated with activation of distinct populations of brain cells.

## Discussion

Female Bengalese finches select their mates based at least primarily on properties of the songs performed by male suitors. The mate preferences of individual females are stable across intervals ranging from hours to months, and establishing a preference for one song but not another does not require that the female has ever had any interaction with the associated male singers. These data do not exclude a role for more general experience in shaping the features of mate preference, however they make clear the importance of the signal in communicating not only the identity of the singer but also the subjective quality of that signal and its sender. Finally, we also found that individual birds could be selective for some song features but not selective for others. The absence of a relation between selectivity for different facets of male song suggests that evaluation of different parameters occurs independently.

Our data forge a link between Bengalese finch sexual preference, evident in a copulation solicitation display, and expression of other overt female behaviors, such as calling in response to hearing a song that the female finds attractive. In some cases, behavioral measures such as hopping onto a perch to evoke song playback (for example, [4]) are used to measure female preference, however those behaviors can leave uncertainty about whether females are engaging with those stimuli because of familiarity or because of a sexual preference for those singers. Other behaviors, such as egg laying [23] or collecting nest materials [28], have also been used as measures of mate preference (for Bengalese finches, see [28]), and the relevance of these behaviors to reproduction is certainly more evident than the relevance of hopping onto a perch. However, the expression of a copulation solicitation display leaves no doubt about the functional relevance of that behavior [6]. Our findings reveal that the song that evoked the greatest number of CSDs also invariably evoked the greatest number of calls, and there was generally good agreement between the number of calls and the number of CSDs associated with the intermediately-preferred and least-preferred song types. Although female songbird mate choice has been studied in a number of different species (reviewed in [36], [42]), it has been quite challenging to identify behavioral indices of mate choice that do not require hormonal manipulation (for example [43]) or experimental settings much larger than those commonly found in the laboratory (for example [44]). Our identification of calls as an index of female BF mate choice (for canaries, see [33]) will enable us to assay female mate preference without the invasiveness or other potential complications associated with estradiol implants. Calls have the additional advantages that there is no need to wait for the onset of hormone effects, there is no concern regarding possible changes due to prolonged hormone exposure, and calls are nearly ubiquitous among subjects as opposed to the relative rarity of CSDs [33], [45], [46]. Together with these benefits, the findings in the present study will enable us to use calls to probe the properties of female BF mate choice and the features of male song that are salient in that evaluation.

Individual female BFs were not identical in their preferences, with many different females identifying different males as their most-preferred suitor. Such individual-specific preferences suggest that rather than being dictated by one dominant song feature, female mate choice is influenced by a suite of features that together define the subjective quality of each stimulus [47]. On the other hand, across the population of birds that we tested there was a trend for 2 males to be more commonly preferred than the other 6 males. The broadly attractive nature of those songs leaves open the possibility that some song features are more influential than others in affecting a female bird’s perception of subjective quality. Thus, the preference of each female reflects both individual-specific features and trends that are broadly shared across the population. Our data reveal that females commonly preferred songs that were performed in the context of a female receiver, and the song changes that occur in that context highlight parameters that may be especially salient in female mate choice. Future experiments to define the relation between changes in those properties and changes in the preference of individual females will provide insight into the forces that have shaped mate selection and the evolution of song characteristics in this species [48].

A particularly intriguing aspect of our dataset is that birds that had not interacted with the males used in these tests report preferences that are very similar to those of females that had interacted with those males. From these data, we can conclude that the subjective value assigned to each song must reflect the quality of the stimulus itself rather than the value of an outcome predicted from previous experience with that singer. Therefore, interaction with a specific male is not necessary for the female to find his song attractive, however this by no means rules out other forms of experience as important factors influencing female mate choice. Studies in other species have revealed that female mate choice is shaped at least partially through experience during a period of early development [49], [50], and an important goal of future experiments will be to define how preferences emerge in the development of individual females. The specificity of the preferences detected here suggests an important role for learning in shaping the detailed characteristics of individual preferences [51], [52], [53], [54], [55]. Because our birds were kept in group-housed cages where no rearing of offspring occurred, females were not able to learn by observing the outcome of mating. Therefore, stimulus value may have been learned by observing the stimulus preferences of other females. Female songbirds that live in large colonial assemblies can recognize individuals based on their calls [56], enabling eavesdroppers to attend to the preferences of specific individuals and suggesting a mechanism through which birds may detect the preferences of other females and use that information to modify their own preferences. An abundance of calling in some cases and a paucity in others, as we observed in our data, may provide a graded measure of preference that is more nuanced than simply the presence or absence of a CSD. The ease of rearing young BF in carefully controlled social contexts opens the door to fascinating investigations of the degree to which female mate preference may be influenced by social interaction with other females (e.g., to observe their preferences) or other males (e.g., to learn a spectrum of performance ability).

Our data also reveal that choosiness for one song facet does not obligate the bird to be choosy for all facets, revealing that multiple song features are evaluated independently. This independence suggests that the mechanisms underlying those aspects of evaluation may include separate populations of neurons, and perhaps even separate brain sites, which participate in detection and evaluation of different facets of song. Specific sites within the auditory forebrain of female songbirds have been implicated in recognition of mate signals [32], [57], [58], [59], and an important future goal will be to couple the behavioral methods used here with high-resolution neurophysiological records to define how preference is encoded in the brain. In such studies, a longitudinal approach will be necessary to define the preferences of each individual, making the consistent preferences of individual BF females an especially valuable trait. In our experience, BFs are the easiest species from which to collect neurophysiological data [60]. Female BFs will provide the additional advantage that individuals vary in the identity of the song they prefer most, and that variation will facilitate disambiguation of how the brain encodes subjective value per se as opposed to the physical properties of the stimulus. Thus, the present results document the mate preferences of adult female BF and establish them as an excellent animal model in which to investigate the mechanisms through which salient features of reproductive signals are detected, evaluated and used to guide the decision of mate choice.
